# Electrocautery Snare Resection and Argon Plasma Coagulation of Endobronchial Kaposi Sarcoma Presenting as an Obstructing Tumor

**DOI:** 10.7759/cureus.5786

**Published:** 2019-09-27

**Authors:** Alfred Papali, Samuel J Minkove, Edward M Pickering, Nirav Shah, Ashutosh Sachdeva

**Affiliations:** 1 Pulmonary & Critical Care Medicine, Atrium Health, Charlotte, USA; 2 Internal Medicine, University of Maryland Medical Center, Baltimore, USA; 3 Pulmonary & Critical Care, University of Maryland School of Medicine, Baltimore, USA

**Keywords:** kaposi sarcoma, endobronchial, bronchoscopy, pulmonary kaposi

## Abstract

Kaposi sarcoma (KS) is the most common neoplasm associated with Acquired Immune Deficiency Syndrome (AIDS), but antiretroviral therapy has reduced its incidence dramatically. Endobronchial KS is usually associated with concurrent mucocutaneous lesions and is highly vascular; so biopsy generally is not recommended. The use of advanced bronchoscopic techniques for evaluation of endobronchial KS may mitigate the bleeding risks but has not been described previously. We describe an unusual case of KS, which presented as an isolated obstructing endobronchial tumor that was effectively resected using electrocautery snare and argon plasma coagulation (APC) during bronchoscopy.

## Introduction

Kaposi sarcoma (KS) remains the most common neoplasm associated with Acquired Immune Deficiency Syndrome (AIDS), but antiretroviral therapy has reduced its incidence dramatically. Endobronchial KS, usually associated with concurrent mucocutaneous lesions, typically appears as slightly raised, sub-mucosal, violaceous lesions in the bronchial wall. Given the vascularity of these lesions, a biopsy is not recommended; however, the use of argon plasma coagulation (APC) or electrocautery may mitigate these risks, making it safer to biopsy and/or ablate the KS lesion. We describe an unusual case of KS, which presented as an isolated obstructing endobronchial tumor that was resected using electrocautery snare and APC during bronchoscopy, followed by cryotherapy ablation of the residual lesion.

## Case presentation

**Figure 1:**
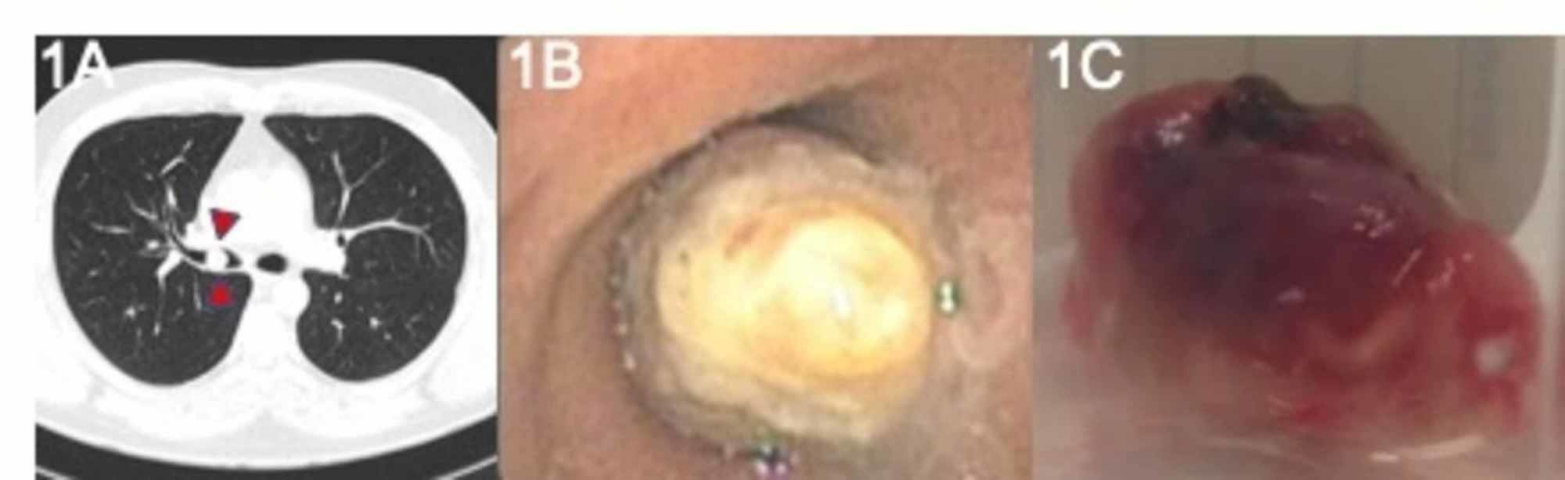
(A) Obstructing mass visualized on CT of the chest (red arrowheads); (B) Obstructing mass visualized in the distal right mainstem bronchus during inspection bronchoscopy; (C) Gross view of mass following resection

A 40-year-old Caucasian male, a non-smoker, was referred for evaluation of an endobronchial lesion diagnosed on thoracic imaging done for workup of four weeks of progressive cough, wheezing, and dyspnea. Past medical history was significant for allergic rhinitis and previously undisclosed Human Immunodeficiency Virus (HIV) for which he had not sought medical attention. Physical examination demonstrated bronchial breath sounds in the right mid-lung zone and no abnormal findings on the skin or oral mucosa. A CT of the thorax revealed a polypoid endobronchial tumor in the distal right mainstem bronchus extending into the bronchus intermedius (BI) without associated parenchymal abnormalities (Figure 1A). Bronchoscopy revealed a near-complete obstructing mass in the distal right mainstem bronchus anchored on a wide stalk (Figure 1B), with otherwise normal-appearing bronchial mucosa throughout the remainder of the tracheobronchial tree. After obtaining fine needle aspiration sampling, an electrocautery snare (ConMed, Utica, NY) was performed to resect the mass (Figure 1C).

**Figure 2:**
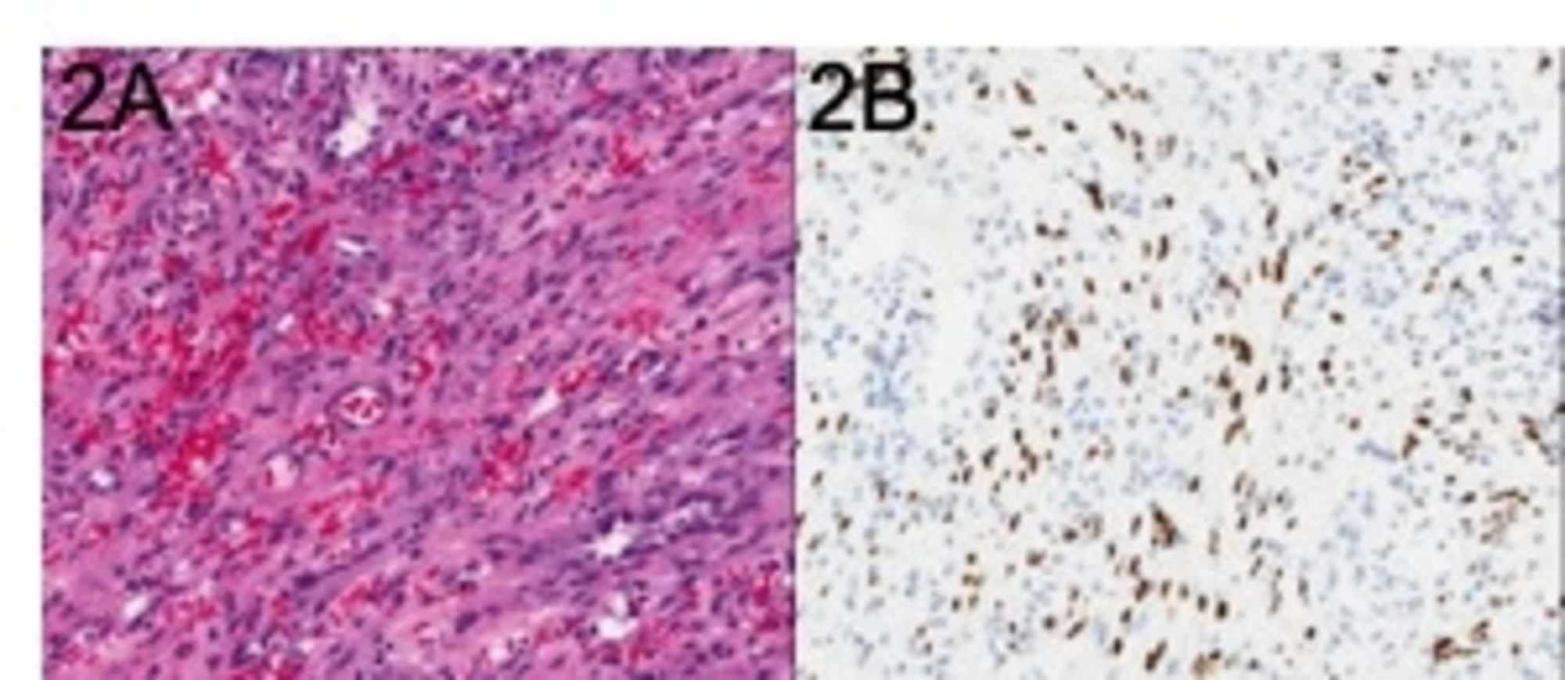
(A) Hematoxylin and eosin stain showing characteristic features of KS; (B) Special staining for human herpesvirus 8 reveals numerous infected cells KS = Kaposi sarcoma

Mild bleeding was well-controlled using APC. Cytologic examination revealed spindle-cell proliferation with mild cytologic atypia (Figure 2A). Immunohistochemical staining for human herpesvirus 8 was positive (Figure 2B), confirming the diagnosis of KS. An absolute cluster of differentiation four (CD4) blood count was 78 per microliter.

**Figure 3:**
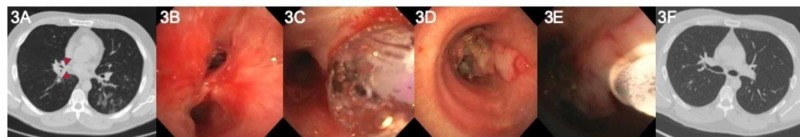
(A) Repeat CT showing partial re-obstruction of the distal bronchus intermedius (red arrowheads); (B) Image of the superior segment on follow-up bronchoscopy; (C) Balloon catheter inflation to re-establish airway patency; (D) Bronchial wall defect visualized in the BI during repeat bronchoscopy; (E) Cryoprobe® removal of excess airway tissue. APC used subsequently to achieve hemostasis; (F) CT imaging six months after bronchoscopic treatment BI = Bronchus intermedius; APC = Argon plasma coagulation

Two days later, a repeat bronchoscopy was performed due to newly reported fever and abnormal CT imaging (Figure 3A). Inspection demonstrated partial obstruction of the BI lumen with devitalized tissue (Figure 3B). Balloon dilation (Figure 3C) using an 8-9-10 pulmonary CRE balloon (Boston Scientific, Natick, MA) was performed. A 5-mm defect in the lateral wall of the distal BI, likely from previous electrocautery (Figure 3D), was visualized but did not require any intervention. Subsequent cryodebulking of devitalized tissue using a 2.4-mm Cryoprobe (Erbe, Germany) resulted in significant improvement in lumen size (Figure 3E). A follow-up bronchoscopy eight weeks later, after initiation of antiretroviral therapy, revealed airway patency and well-healed bronchial tear without evidence of KS recurrence. The patient enrolled in a clinical trial for the treatment of his KS. Initial whole-body positron emission tomography (PET) scan revealed no tracer uptake in the thorax and repeat chest CT six months after therapy demonstrated normal airway anatomy (Figure 3F).

## Discussion

Endobronchial lesions associated with both endemic and immunosuppression-related KS are well-documented. The vast majority of them appear as flat-to-slightly-raised violaceous lesions, often located near minor carinae [1]. Differential diagnosis includes infections from fungi, Bartonella (bacillary angiomatosis), and mycobacterium, as well as malignancies, including lymphoma and squamous cell cancer. Concurrent skin and oral mucosal KS lesions are the norm, but endobronchial findings in the absence of lesions elsewhere on the body are uncommon [2]. Nodular and mass-like lesions have also been described [3]; however, endobronchial KS presenting as an obstructing mass without concurrent mucocutaneous disease had been reported only twice previously [4,5].

This case is unique both in its unusual presentation and its use of therapeutic bronchoscopic techniques not previously described for the treatment of endobronchial KS. Despite the unknown nature of the tumor at the initial presentation, its polypoid nature made it amenable to snare electrocautery resection. Its wide pedicle, suggesting endobronchial invasion, necessitated cryotherapy and subsequent APC to achieve hemostasis and to destroy residual malignant tissue. We chose electrocautery and APC because of its ease, good safety profile, the similar risk of iatrogenic endoluminal damage between electrocautery and Nd:YAG laser [6], and equivalency in re-establishing airway patency compared to laser [7]. Early reports of endobronchial KS cautioned against biopsy due to high bleeding risk [8], but others have suggested that this complication is relatively infrequent [9]. In our case, bleeding risk was minimized with the use of snare electrocautery, given simultaneous cauterization of the base of the tumor during resection. Further treatment of endobronchial tumors usually requires follow-up procedures to manage granulation tissue, to confirm the resolution of the disease and, as in our case, to address post-electrocautery scarring.

## Conclusions

Kaposi sarcoma presenting as a localized, obstructing airway mass without concurrent mucocutaneous disease is extremely rare but should be considered in the differential diagnosis in patients with known immunocompromised state. While tumor manipulation risks bleeding, advanced endoscopic techniques in the hands of trained operators allow for safe and effective intervention.

 

This article was originally published as an abstract: Papali A, Sachdeva A, Pickering E, Shah N: Electrocautery Snare Resection and Argon Plasma Coagulation of Endobronchial Kaposi Sarcoma Presenting as an Obstructing Tumor. Chest 2014, 146:7773A. 10.1378/chest.1993118

## Appendices

AP drafted the original manuscript. SJM revised the manuscript and obtained patient consent. EMP, NGS and AS reviewed the manuscript for content. All authors read and approved the final manuscript.
